# Significant improvement of psychotic symptoms in treatment-resistant schizophrenia with clozapine in an adolescent with SHINE syndrome: a case report

**DOI:** 10.1186/s12888-023-04962-y

**Published:** 2023-06-29

**Authors:** Maxine Yang, Arielle Rubin, Rebecca Wondimu, Theresa Grebe, Gaby Ritfeld

**Affiliations:** 1 University of Arizona College of Medicine – Phoenix, Phoenix, AZ 85004 USA; 2 Phoenix Children’s Hospital, Phoenix, AZ 85016 USA

**Keywords:** Synaptopathy, Clozapine, Early-onset, Treatment-resistant, Schizophrenia, Psychosis, Genetic testing, Pediatric

## Abstract

This report highlights a rare single-gene cause of early-onset, treatment-resistant schizophrenia, and its unique responsiveness to clozapine therapy. This case describes a pediatric female who was diagnosed with early-onset schizophrenia and catatonia in her early adolescence, and was later found to have DLG4-related synaptopathy, also known as SHINE syndrome. SHINE syndrome is a rare neurodevelopmental disorder caused by dysfunction of the postsynaptic density protein-95 (PSD-95), encoded by the DLG4 gene. After failing three antipsychotic drug treatments, the patient was started on clozapine, which resulted in significant improvements in positive and negative symptoms. This case illustrates the impact of clozapine in treatment-resistant early-onset psychosis and exemplifies practical implications for genetic testing in early-onset schizophrenia.

## Background

Early-onset schizophrenia (EOS), schizophrenia with onset before 18 years old, Very Early Onset Schizophrenia (VEOS), schizophrenia with onset before 13 years old, and psychotic symptoms in pediatric patients are rare and require further workup to exclude primary psychiatric and neurologic conditions, infectious processes, and toxic or metabolic causes. While adult-onset schizophrenia has been studied in detail, research on EOS is limited, partly due to its low prevalence and because EOS was not recognized in the diagnostic systems before the introduction of DSM-III in 1980. The prevalence of schizophrenia in children and adolescents is low, about 1.4 in 10,000 before the age of 15 [1, 2]. The etiology of EOS is not well understood because of the scant data on this condition, and even less is known about the role of genetic variations in EOS, which leads to the low incidence of genetic testing in patient workup unless the patient’s family history includes schizophrenia [3]. One rare genetic cause of EOS is SHINE syndrome.

SHINE syndrome, also known as DLG4-related synaptopathy, is a neurodevelopmental disorder due to dysfunction of the postsynaptic density protein-95 (PSD-95), encoded by DLG4. The prevalence of this disorder is unknown, with 53 patients confirmed in global literature reviews [4]. PSD-95 regulates excitatory synaptic function, strength, and plasticity in the brain. It plays a key role in brain development and function with major modulatory effects on glutamatergic synapses. Dysfunction in these receptors alters excitatory synaptic transmission, leading to positive, negative and cognitive symptoms. Developmental regression is also a common symptom amongst SHINE patients. In addition, several behavioral issues including self-aggression, behavioral outbursts, bipolar disorder, catatonia, and obsessive–compulsive disorder (OCD), have been described in conditions that affect glutamatergic neuron activity [5, 6].

SHINE syndrome is an acronym for a common set of symptoms seen in affected patients, including Sleep disturbances, Hypotonia, Intellectual disability, Neurological disorders, and Epilepsy [7]. Autistic features, regression in motor and language skills, and psychosis have been reported in less than half of cases. Psychosis is not considered a cardinal feature of the condition. Our understanding of the relationship between SHINE syndrome and early onset psychosis is limited, with fewer than 10 documented cases in the literature.

## Case presentation

An early-adolescent female with a past medical history of learning disability diagnosed in 1^st^ grade, presented to the psychiatry clinic with a 7-month history of selective mutism and depressed mood.

She had an unremarkable birth history and had met her motor milestones. She did not start speaking until 23 months, when she reportedly started speaking in age-appropriate full sentences. In addition, she was reported not to smile or interact as much as other children but was still “a happy and social child and made friends”. Her mother reported that she was diagnosed with a learning disability in 1^st^ grade and placed on an Individualized Education Plan (IEP) for math and reading, but excelled in those classes with A’s and B’s. At the time of the initial visit, she had recently started 8^th^ grade. She enjoyed gymnastics, tumbling, and cheerleading until October of 2019. Menarche occurred at 13 years old, and menses were irregular, with only 8 periods from 2019–2021. She has a family history of bipolar I with psychotic features in her paternal grandmother that responded well to quetiapine. Otherwise, family psychiatric history was negative. She lives with her parents and 18-year-old brother and has no history of tobacco, alcohol, or drug use.

At the initial visit, the patient softly told her parents that “The Voice” told her not to talk. She was diagnosed with selective mutism and adjustment disorder at presentation, started on sertraline and Cognitive Behavioral Therapy (CBT). She continued to withdraw with ongoing refusal to speak to family and peers. She began whispering to internal stimuli and displayed physical aggression to her parents. Within a few months, she stopped participating in self-care/hygiene and developed mixed catatonia, with mutism, posturing, and agitation. She was hospitalized and worked up for new-onset psychosis with EEG, MRI, CT Abdomen, CT Chest, CT Pelvis, and CSF studies with encephalitis panel, lysosomal enzyme panel, CRP, ESR, and TPO antibody tests. All results were normal, and she was started on clonazepam and risperidone. Risperidone 1 mg worsened her mutism, stereotypical movements, and odd behaviors, and she stopped eating and drinking. The risperidone was stopped while the clonazepam continued. During this time, she minimally interacted with others, and still responded to internal stimuli. There was minimal improvement in her symptoms with clonazepam, but she was unable to tolerate increased doses as it made her louder and more difficult to manage. Decreasing clonazepam made her less verbal, more withdrawn, and agitated. Oanzapine 5 mg was added with improvements in aggression and was discharged with residual catatonia. However, at home she would pace the house for hours, and frequently tried to elope. She had a good appetite but was still frequently responding to internal stimuli. Her catatonia improved, and clonazepam was tapered off. Olanzapine was increased with mild improvements in speech production, and she was able to answer questions with one to two-word responses. However, she was reported to start throwing objects and screaming daily, particularly at night, which interfered with her sleep. Her appetite increased and her weight subsequently increased. She was diagnosed with early-onset schizophrenia in March 2021. In June 2021, due to olanzapine’s metabolic side effects, she began metformin treatment to help with weight gain, hyperlipidemia and elevated HgA1c.

In November 2021, she started on a cross-titration from olanzapine 7.5 mg daily to aripiprazole 20 mg daily due to ongoing internal preoccupation and psychotic symptoms. She also presented with significant side effects of Parkinsonism and had a 30 lb weight gain on olanzapine, limiting further titration. Although cross-titration to aripiprazole helped her lose 5 lbs and normalized her appetite, she began to have insomnia, regression of speech, and worsening episodes of physical aggression. In March 2022, she was restarted on olanzapine, which stabilized her aggression but resulted in another 10 lb weight gain. She was still responding to internal stimuli and displayed prominent negative symptoms.

She was evaluated in genetics clinic where she was noted to be normally grown and minimally dysmorphic, with hypertelorism and a prominent nose. Her early development history suggested that an underlying genetic neurodevelopmental syndrome was responsible for her psychosis. The differential diagnosis included many chromosome disorders, most prominently 22q11.2 deletion syndrome. Neurometabolic disorders other than Wilson disease, were also of concern as many are treatable. Genetic testing was ordered, including a chromosomal microarray analysis (CMA) and whole exome sequencing. The CMA was normal but the whole exome sequencing revealed a de novo mutation in the gene DLG4, consistent with DLG4-related synaptopathy also known as SHINE syndrome. Specifically, she carries thep. (Arg660Ter) (CGA > TGA): c. 1978 C > T in exon 19 of the DLG4 gene which is a nonsense variant that is predicted to result in protein truncation or decay. This variant has been observed de novo without confirmed parentage in multiple unrelated patients with DLG4-related neurodevelopmental disorder in the published literature [4]. The patient also carries a likely pathogenic variant in the MT-TA gene at a 2% heteroplasmy level. Due to the low level of heteroplasmy, it is unknown whether she will exhibit symptoms of a mitochondrial disorder, although the typical lower threshold for the presence of any mitochondrial disease symptoms is 5% heteroplasmy.

In May 2022, her parents consented to clozapine treatment due to her treatment-refractory schizophrenia, having trialed risperidone, aripiprazole, and olanzapine. Metformin was increased to 2000 mg daily, and polyethylene glycol was started. Baseline studies for clozapine safety monitoring were obtained. Troponin, ANC, and CRP were within normal limits. EKG showed concern for Wolff-Parkinson-White (WPW) syndrome, and after subsequent Holter monitoring for 30 days, she was cleared by cardiology in July 2022 to begin clozapine therapy. Because of the prevalence of autism spectrum disorder (ASD) in SHINE syndrome, she was referred for a formal autism evaluation and psychological testing. Her mother had suspected she had high-functioning ASD since she was 3 years old as she struggled to relate socially to children of the same age. At the time of testing, she demonstrated moderate to severe intellectual disability and was diagnosed with co-morbid ASD.

To objectively measure the patient’s improvement while on clozapine, the Vineland Adaptive Behavior Scales (VABS) (Vineland-3) Comprehensive Interview Form was used as a reference to assess parent-reported changes in adaptive functioning. The VABS is a measure of adaptable behavior used to assess individuals with intellectual and developmental disabilities by scoring the patient’s adaptive behavior abilities in the domains of communication, daily living, and socialization. A baseline assessment was obtained prior to clozapine initiation, and she was scored again 8 months into the clozapine treatment, to track her improvements. Her VABS Composite score ranked below the first percentile throughout treatment, however, she showed improvements in raw scores across multiple subdomains, consistent with newly acquired skills while on clozapine.

Improvements in the daily living skills subdomain included acquiring self-sufficiency in areas of feeding, dressing and hygiene, with the personal daily living skills raw score improving from 81 to 87. Her interpersonal relationships, part of subdomain socialization, demonstrated improvements in social appropriateness, behavioral and emotional control, with a raw score increase from 18 to 22. Receptive language raw score improved from 43 to 51, and included improved ability to follow instructions and improved attention. Written skills raw score improved from 29 to 34, with greater written vocabulary and word recall from memory. Her expressive language skills remained unchanged, with pre- and post-clozapine treatment scores at 23. Her domestic skills raw score improved from 14 to 20, with the patient now able to make her bed daily and prepare simple meals for herself.

Her mother reports that the patient seems to experience decreased frequency, intensity, and duration of hallucinations. She is less internally distracted and more engaged socially, wanting to participate in family activities and able to participate in testing to return to in-person school. She has been experiencing a broader range of affect since the clozapine treatment, with more frequent smiling. Her sleep and appetite have also normalized.

The patient was monitored for clozapine side effects and toxicity weekly for the first 6 months and biweekly thereafter. While on clozapine, the patient experienced increased abdominal girth, which is a well-documented side effect of clozapine [8]. She did not experience other metabolic side effects. She did experience sedation during initial titration, which resolved. During the initial months of treatment, the patient exhibited tachycardia of approximately 115 beats per minute. As the clozapine dose was increased above 200 mg, the patient's heart rate rose to 130 beats per minute, with associated symptom of fatigue. Following extensive evaluation that included EKG, echocardiogram, serum troponin and CRP monitoring, and cardiology consult, the tachycardia was attributed to the use of clozapine, which is a known side effect of the atypical antipsychotic [8]. The patient was started on atenolol 12.5 mg daily, which resolved the tachycardia and alleviated the associated fatigue. The patient was prophylactically prescribed polyethylene glycol as a stool softener prior to the onset of clozapine titration, and had no reported constipation during treatment. The patient did not experience orthostatic hypotension, myocarditis, alterations in absolute neutrophil count or other blood cell counts, extrapyramidal symptoms, sialorrhea, or other side effects. Overall, the patient’s side effect profile on clozapine appears similar to those of patients without SHINE syndrome.

## Discussion

This report alerts health professionals to the existence of SHINE syndrome and the need to include it in the differential diagnosis for acute psychotic symptoms in children. We also call for more research on synaptopathies to better understand and treat this disease. SHINE syndrome, also known as DLG4-related synaptopathy, is a neurodevelopmental disorder due to dysfunction of the postsynaptic density protein-95 (PSD-95), encoded by DLG4. The prevalence of this disorder is unknown, with only 53 patient cases in global literature reviews [4]. PSD-95 regulates excitatory synaptic function, strength, and plasticity in the brain. It plays a key role in brain development and function with major modulatory effects on glutamatergic synapses. Dysfunction in these receptors leads to altered excitatory synaptic transmission. PSD-95 has a unique role in uncoupling dopamine-glutamate and dampening the interaction between them. Malfunction of this receptor may result in concomitant overactivation of both D_1_ and N-methyl-D-aspartate (NMDA) receptors, jeopardizing neuronal integrity and triggering neurotoxicity. An important consequence of the failure of this feedback loop is increased dopamine levels in the mesolimbic and mesocortical dopamine pathways, leading to positive, negative and cognitive symptoms, respectively [5, 8].

Our case study shows that SHINE syndrome may present with treatment-resistant psychosis in youth. Our patient’s treatment-resistant schizophrenia secondary to SHINE syndrome is a rare presentation and is also uniquely responsive to clozapine therapy. It should be noted that the patient does have a family history of a paternal grandmother with adult-onset bipolar disorder with psychotic features. However, both her parents, including her father, as well as extended family members, are free from psychosis and other psychiatric symptoms. This suggests overall low genetic loading for typical psychotic illness. Moreover, unlike her grandparent's illness, the patient's psychosis was early-onset, associated with profound regression and was initially treatment resistant. Additionally, while psychosis is not considered a cardinal feature of SHINE syndrome, 10% of the 53 documented cases had reported psychosis [4]. Therefore, although an interaction between genetic factors cannot be excluded, it is highly likely that this child’s mutation related to SHINE syndrome is the most significant contributing cause of her psychosis.

We hypothesize that our patient's treatment-resistant psychosis was related to the NMDA dysfunction secondary to SHINE syndrome, and therefore responsive to glutamatergic modulation using clozapine. Clozapine is the gold standard for treatment-resistant schizophrenia. Prior studies have found neurons from clozapine-responsive patients that exhibited a reciprocal dysregulation of gene expression, particularly related to glutamatergic and downstream signaling, which was possibly reversed by clozapine treatment. Clozapine treatment decreased the intensity and distress of internal stimuli, affective lability, aggression, social withdrawal, isolation, thought blocking, world salad, and affect blunting in our patient. In addition, the Vineland Adaptive Behavior Scale showed notable improvements in executive function, receptive language, daily living skills, and socialization. Understanding the unique pathophysiological mechanisms of psychosis in SHINE syndrome is necessary to better predict who is at risk for treatment-resistant psychosis and when to consider clozapine. This case also highlights the importance of interdisciplinary care in pediatric patients with psychosis by illustrating the critical role of genetic specialists in providing specific diagnoses which thereby facilitate appropriate medical treatment.

Currently, treatment strategies due to genetic variants for psychosis remain limited. Our decision to use clozapine as a treatment for resistant schizophrenia was aided by the knowledge of the patient’s SHINE syndrome diagnosis, due to a mutation in synapse proteins related to glutamate. The ability of clozapine to modulate glutamatergic activity made it particularly suitable for this case. Identification of the glutamate protein mutation helped build our understanding of other mechanisms, and ultimately enabled us to arrive at the treatment decision for clozapine for this patient. These results can provide information regarding how genetic changes affect neural pathways and proteins, consequently furthering research capabilities in creating unique interventions and personalized care. This can help to bridge the knowledge gap present today, leading to more tailored health care solutions for those living with SHINE syndrome and other genetic neurodevelopmental disorders.

## Conclusion

This study illustrates the role of SHINE syndrome in the differential diagnosis for acute psychotic symptoms in pediatric patients, and how clozapine can be used as a treatment, especially for the positive and negative cognitive symptoms domains of psychosis secondary to SHINE syndrome. By describing this unique presentation of SHINE syndrome, we hope to contribute towards appropriate clinical suspicion, to disseminate knowledge of the existence of this condition, and to call for studies on the possible endocrinologic effects on this disease process. This report also highlights the importance of genetic screening within the diagnosis of Early Onset Schizophrenia. Lastly, the inclusion of genetic testing within the work up of EOS could provide researchers with new treatment possibilities, improving the overall prognosis of affected individuals.

## Data Availability

All data generated or analyzed during this study are included in this published article.

## Abbreviations

SHINE: Sleep disturbances, Hypotonia, Intellectual disability, Neurological disorders, and Epilepsy
PSD-95: Postsynaptic Density Protein-95
EOS: Early-onset Schizophrenia
VEOS: Very Early Onset Schizophrenia
DSM: Diagnostic and Statistical Manual of Mental Disorders
OCD: Obsessive–Compulsive Disorder
IEP: Individualized Education Plan
CBT: Cognitive Behavioral Therapy
CMA: Chromosomal Microarray Analysis
WPW: Wolff-Parkinson-White
ASD: Autism Spectrum Disorder
NMDA: N-Methyl-D-Aspartate
VABS: Vineland Adaptive Behavior Scales
